# The histological and radiological evaluation of autologous peripheral venous blood concentrates in socket preservation: a systematic review and meta-analysis

**DOI:** 10.3389/fdmed.2025.1602738

**Published:** 2025-07-11

**Authors:** Yusheng Meng, Min Wu, Shuang Wang, Xiuqiao Yang, Yun Liu

**Affiliations:** Stomatology Health Care Center, Shenzhen Maternity and Child Healthcare Hospital, Southern Medical University, Shenzhen, Guangdong, China

**Keywords:** autologous peripheral venous blood concentrates, platelet-rich plasma, platelet-rich fibrin, concentrated growth factor, socket preservation

## Abstract

**Objectives:**

A detailed meta-analysis and systematic search was conducted to assess the histologic and radiographic efficacy of autologous peripheral venous blood concentrates (APVBCs) for the socket preservation.

**Design:**

Electronic databases were searched until 31 January 2025. Randomized controlled trials (RCTs) and controlled clinical trials (CCTs) in English were identified. Alveolar bone reconstruction was assessed through histologic and radiographic evaluation after tooth extraction. Data were analyzed using Revman5.3, and the risk of bias was evaluated with the Cochrane Collaboration tool.

**Results:**

A total of 16 studies (12 RCTs and 4 CCTs) involving 619 sites was included in our meta-analysis. The results indicated that the APVBCs application significantly reduced the vertical bone resorption in the buccal ridge (standardized mean difference [SMD]: −0.30; 95% confidence interval [CI]: −0.54 to −0.06; *p* = 0.02, *I*^2^ = 5%) and palatal/lingual ridge (SMD:−0.30, 95% CI: −0.54 to −0.06; *p* = 0.02, *I*^2^ = 0%) by radiographic analysis. In addition, the vertical resorption of the buccal and palatal/lingual alveolar ridge was significantly reduced by using materials combined with APVBCs as the filling material for extraction sockets. The newly formed bone percentage showed a statistically significant increase in APVBC presence during socket preservation (SMD: 1.27, 95% CI: 0.65–1.89; *p* < 0.0001, *I*^2^ = 71%) and APVBC + material groups (SMD: 0.85, 95% CI: 0.35–1.35; *p* = 0.0009, *I*^2^ = 0%). However, APVBCs + materials did not show significant effects on the remaining graft particles.

**Conclusions:**

APVBCs in socket preservation can reduce vertical bone resorption and enhance new bone formation. Meanwhile, APVBCs may improve osteogenic efficiency with bone graft material.

**Systematic Review Registration:**

https://www.crd.york.ac.uk/PROSPERO/view/CRD420250653020, identifier CRD420250653020.

## 1 Introduction

Dental extraction is a common procedure in oral clinical practice. However, it often triggers an inflammatory response that leads to alveolar ridge bone resorption. After extraction, 40%–60% of alveolar bone may be lost horizontally and vertically, which negatively affects subsequent implant placement and prosthetic treatment (1). In addition, the thickness of the keratinized gingiva and soft tissue is also reduced due to alveolar bone resorption.

Socket preservation (SP) can help reduce alveolar ridge bone resorption and address associated soft tissue issues. Initially proposed in 1974, socket preservation refers to protective intervention at sites requiring delayed implant placement (2). It is designed to minimize the risk to existing tissue and create favorable conditions for new bone formation (3).

To maintain the alveolar ridge and gingival contour, various hard and soft tissue preservation techniques and materials are available, such as autologous peripheral venous blood concentrates (APVBCs), bone grafts, and substitutes. Bone grafts and substitutes, including autogenous, xenografts, allografts, growth factors, or stem cells, provide the mechanical support necessary for socket preservation (4).

APVBCs are derived from centrifuged autologous peripheral blood. To date, three generations have been developed: platelet-rich plasma (PRP), platelet-rich fibrin (PRF), and concentrated growth factor (CGF) (5). PRP, the first generation, was described by Whitmen et al. and is a platelet-rich plasma obtained by centrifuging the patient's peripheral venous blood with anticoagulants. It as commonly used as a biochemical aid in oral surgery (6). PRF, the second generation, was introduced by Chouckroun et al. in 2000 and is prepared by centrifuging blood without anticoagulants (7). CGF, the most recent generation, was first reported by Sacco in 2006. Like PRF, it is obtained by centrifugation, but with a different protocol that yields a denser fibrin network containing higher concentrations of cytokines (Medifuge, Silfradent, Italy) (8). The differences among APVBCs are shown in Table 1.

**Table 1:** The differences in APVBC characteristics.

The classification of APVBCs	First reported	The preparation protocol	Anticoagulants	The separation layers of APVBCs after centrifugation	Function
PRP	Whitman in 1997	5,600 rpm then red blood cells (RBCs) and buffy coat for 2,400 rpm	Citrate–phosphate–dextrose solution	Top layer: PPP. Middle layer: PRP Bottom layer: RBC	Wound healing; release the growth factors
PRF	Choukroun in 2000	3,000 rpm for 10 min	No	Top layer: PPP. Middle layer: PRF. Bottom layer: RBC	Wound healing; Osteogenicity; release the growth factors and cytokines
CGF	Sacco in 2006	Acceleration for 30 s, 2,700 rpm for 2 s, 2,400 rpm for 4 s, 2,700 rpm for 4 s, 3,000 rpm for 3 s and deceleration for 36 s	No	Top layer: PPP. Middle layer:: CGF and Buffy coat Bottom layer: RBC	Wound healing; Osteogenicity; release the growth factors and cytokines; 3D fibrin network structure

PPP, platelet-poor plasma; PRP, platelet-rich plasma; RBC, red blood cell; PRF, platelet-rich fibrin; CGF, concentrated growth factor.

APVBCs, as a biomaterial containing a range of growth factors and cytokines, can be easily obtained from the patient's blood and used to fill alveolar ridge bone defects, either as a membrane or a gel-like substance, as shown in some existing research (9). These preparations contain various growth factors and have a unique fibrin network structure that enables stable attachment of more cytokines. Platelet-derived growth factor (PDGF), transforming growth factor β (TGF-β), bone morphogenic proteins (BMPs), and others promote tissue regeneration and wound healing by accelerating the migration and proliferation of mesenchymal stem cells (10). providing nutrients and oxygen for new bone formation (11), and stimulating the Wnt/β-catenin signaling pathway to induce osteogenic differentiation of bone marrow stromal cells (BMSCs) (12). The fibrin lattice structure of the APVBCs exhibits both osteoconductive and osteoinductive properties. Some clinical trials have demonstrated their potential benefits for socket preservation (13). APVBCs may also positively influence the long-term survival and success of dental implants when used in conjunction with socket preservation (14).

However, research on the use of APVBCs alone or in combination with bone substitutes for socket preservation remains limited. Notable differences have been observed between the use of APVBCs alone and their combination with bone substitutes in terms of changes to alveolar ridge morphology and structure. Therefore, the aim of this systematic review and meta-analysis was to evaluate the histological and radiological effects of APVBCs – used alone or with bone substitutes – on alveolar contour changes, newly formed bone, and remaining graft particles in socket preservation.

## 2 Materials and methods

This systematic meta-analysis was conducted based on the Cochrane Handbook guidelines and in compliance with the Preferred Reporting Project Items for Systematic Reviews and Meta-Analyses (PRISMA) statement (15). This study was registered in the PROSPERO database (registration no. CRD420250653020).

### 2.1 PICOS criteria

The inclusion criteria for this systematic review are as follows:

Patients (P): adult patients undergoing socket preservation after tooth extraction, in good physical and mental health, without systemic medical conditions or contraindications for socket preservation.
Intervention (I): use of autologous peripheral venous blood concentrates (APVBCs) during socket preservation.
Comparison (C): socket preservation without the use of autologous peripheral venous blood concentrates (APVBCs).
Outcome (O): vertical and horizontal bone resorption evaluated radiographically; percentage of new bone formation and remaining graft particles assessed through histomorphometric analysis.
Study design (S): randomized controlled trials (RCTs) or controlled clinical trials (CCTs).

### 2.2 The exclusion criteria

The exclusion criteria were as follows: (1) patients with systematic conditions affecting oral diseases and/or those who were pregnant; (2) case reports, animal studies, in vitro studies, reviews, and non-randomized controlled trials; (3) studies evaluating third molars after extraction sockets; and (4) no outcome of interest and insufficient information about socket preservation procedures.

### 2.3 Information sources and data collection

An electronic search of articles in English was conducted across five databases: PubMed, Embase, Cochrane Library, Web of Science, and Scopus. The search included studies published in English from inception to 31 January 2025. The following search terms were used: ((((((blood platelets[Title/Abstract]) OR (platelet rich plasma[Title/Abstract])) OR (platelet-rich fibrin[Title/Abstract])) OR (leukocytes[Title/Abstract])) OR (platelet-rich fibrin[Title/Abstract])) OR (concentrated growth factor[Title/Abstract])) AND (((((alveolar ridge preservation) OR (alveolar bone repair)) OR (socket preservation)) OR (socket healing)) OR (socket regeneration)).

Titles and abstracts from the search were independently screened by two reviewers (Wang and Yang). Full-text articles meeting the inclusion criteria were then reviewed, and any disagreements regarding inclusion or exclusion were resolved through open discussion between the two reviewers. The accuracy of the data was verified by the other reviewers (Liu and Wu).

The following data were collected from each study: author, title, publication year, study design (including number of patients and sites, age and gender of participants, and intervention), procedural methods, and measurement methods of outcomes. The main outcomes were bone resorption evaluated by cone-beam computed tomography (CBCT) and new bone formation assessed through histomorphometric analysis of bone biopsies.

### 2.4 Assessment of risk of bias

The risk of bias for the included RCTs or CCTs was assessed using the Cochrane Risk of Bias (ROB 2) tool. Two reviewers (Wu and Wang) independently evaluated the risk of bias based on the Cochrane Handbook for Systematic Reviews of Interventions. Any disagreement was resolved by open discussion, and consensus was achieved. The considered items included the following: (1) random sequence generation, (2) allocation concealment, (3) blinding of participants and personnel, (4) blinding of outcome assessment, (5) incomplete outcome data, (6) selective outcome reporting, and (7) other bias. The risk of bias in the included studies was classified into three categories: (1) low risk of bias: all items were rated as low risk, or only one criterion was inadequate; (2) moderate risk of bias: two or more items were inadequate, but none were at high risk; (3) high risk of bias: one or more criteria were rated as high risk of bias. Cochran’s Q test and the *I*^2^ statistic were used to assess heterogeneity among the studies.

### 2.5 Statistical analysis

The data analysis was performed using Review Manager 5.3 (Cochrane Collaboration, Oxford, UK). Continuous data from the included studies were reported as a mean difference (MD) or standardized mean difference (SMD) and 95% confidence interval (CI). The meta-analysis for studies with similar group comparisons and a descriptive summary was provided for studies unavailable for meta-analysis. When heterogeneity between the studies was low (*I*^2^ ≦ 50%), the fixed effect model (FE) was applied to the meta-analysis, and when heterogeneity between the studies was significant (*I*^2^ > 50%), the random-effects model (RE) was used. Heterogeneity between studies was assessed for both the radiographic and immunohistochemical analyses. In addition, the corresponding forest plots were generated.

## 3 Results

### 3.1 Study selection and characteristics

**Figure 1:**
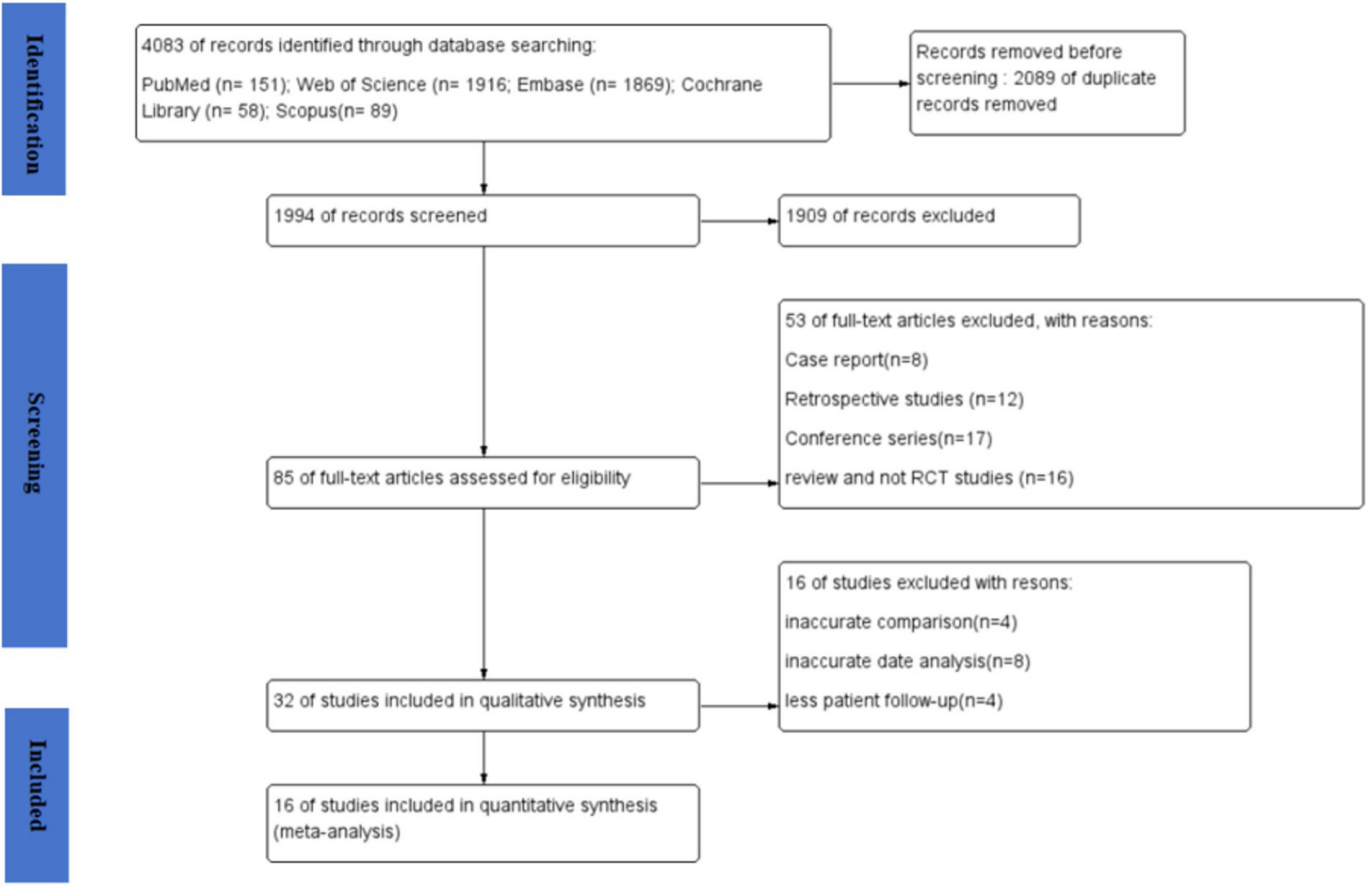
Flow diagram for the article selection process.

A total of 4,083 articles were obtained in the online search as follows: 151 from PubMed, 1,916 from Web of Science, 1,869 from Embase, 58 from Cochrane Library, and 89 from Scopus. In total, 1,994 records remained after excluding any duplicates. Subsequently, 85 articles underwent a thorough full-text review, from which 16 articles were finally included in the meta-analysis (16–31). The flow diagram for the article selection process is shown in Figure 1. The included studies were conducted in the following countries: China (five studies), Egypt (two studies), Spain (one study), Belgium (one study), Lithuania (one study), Brazil (one study), Iran (one study), Thailand (one study), India (one study), and the USA (one study). Regarding study design, two studies (21, 29) used a split-mouth design and 14 studies used a parallel-design design. The regions of tooth extraction were primarily single-rooted teeth, including incisors, canines, and premolars. The types of APVBCs used in the studies were as follows: 12 studies used PRF, three studies used CGF, and one study used PRP. In total, 619 extraction sites were reported across the included studies. Of these, 208 sites were treated with AVPBCs to fill the extraction sockets, and 207 sockets underwent spontaneous healing and served as the control. In addition, 102 sockets were treated with a combination of AVPBCs and graft materials, while another 102 sockets received graft materials only. The main characteristics and outcome data of the included studies are presented in Table 2.

**Table 2:** Characteristics of included studies.

Studies evaluating socket preservation with APVBCs	Year	Country	Type of study	Age	M/F	Duration	Category	Sample size	Groups (number)	Location (number)	Histological and radiological outcome
Amer O	2025	Egypt	CCT parallel-design	35.5 ± 7.9	10/12	6 months	i-PRF	22	T:ADDG + I-PRF (11) C: ADDG (11)	Incisor (7) Canine (3) Premolar (12)	The application of PRF with ADDG were not significantly change in the formed osseous tissues
Mousavi Y	2024	Iran	RCT parallel-design	NG	3/21	8 weeks	L-PRF	24	T: L-PRF (12) C: natural healing (12)	Incisors (2) Canine (9) Premolar (13)	The LPRF neither reduces the rate of ridge resorption in vertical or horizontal dimensions of extraction sockets nor induces more new bone formation
Abaza G	2023	Egypt	RCT parallel-design	30.6 ± 4.5	16/20	4 months	L-PRF	36	T: I-PRF with xenografts (12) HA with xenografts (12) C: xenografts alone (12)	Incisors/Canine (18) Premolar (18)	HA led to improved histological bone maturation.
Abad CE	2023	Spain	RCT parallel-design	57.56 ± 11.17	13/14	4 months	L-PRF	27	T: L-PRF (14) C: natural healing (13)	Incisors (9) Premolar (18)	L-PRF neither minimized bone resorption nor reduced bone regeneration
Wang X	2022	China	RCT parallel-design	41.67 ± 12.73	18/18	5 months	L-PRF	28	T: L-PRF (14) C:natural healing (14)	Incisors (2) Canine (5) Premolar (29)	L-PRF increased the growth factors concentrations
Liu Y	2022	China	RCT parallel-design	31.1 ± 11.38	11/11	6 months	CGF	22	T: DBBM + CGF (11) C:DBBM + Collagen (11)	Incisors (21) Premolar (3)	The application of CGFs in ARP neither minimized bone resorption increased the width of keratinized
Keranmu D	2022	China	RCT parallel-design	28.89 ± 2.7	15/23	3, 6 months	CGF	38	T: DBBM + CGF (19) C:DBBM (19)	Incisors (26) Canine (12)	The CGF combined with DBBM can help to maintain the contour of alveolar bone
Lin S-c	2021	China	RCT parallel-design	48.3 ± 7	21/15	8 months	CGF	36	T1: DBBM + CGFs (12) T2: DBBM (12) C:Natural (spontaneous) Healing (12)	frst molar (27) second molar (9)	The CGF combined with DBBM effectively reduced the bone resorption and resulted in more newly formed bone
Castro AB	2021	Belgium	RCT a split-mouth design	NG	6/15	3 months	PRF	60	T1: L-PRF (20) T2: A-PRF + (20) C:Natural (spontaneous) Healing (20)	central incisors (25), lateral incisors (16): canines: (19)	PRF can help to increase newly formed bone and fail the minimized bone resorption
Stumbras A	2020	Lithuania	RCT parallel-design	T1:48 ± 13 T2: 54 ± 11 T3:43 ± 19 C: 51 ± 14	14/26	3 months	PRGF	40	T1: PRGF (10) T2:BBM/CM (10) T3: FDBA/CM (10) C:Natural (spontaneous) Healing (10)	NG	BBM/CM or PRGF is beneficial to reduce horizontal and vertical bone changes
Canellas JVdS	2020	Brazil	CCT parallel-design	44.8 (18–69)	21/27	3 months	L-PRF	48	T: L-PRF (24) C:Natural (spontaneous) Healing (24)	Incisors (14) Canine (3) Premolar (31)	L-PRF provides significant benefits in terms of alveolar preservation
Areewong K	2019	Thailand	RCT parallel-design	50.67 (22–73)	15/21	2 months	PRF	36	T: L-PRF (18) C:Natural (spontaneous) Healing (18)	NG	PRF in ARP does not statistically significant enhance new bone formation
Zhang YD	2018	China	RCT parallel-design	20–40 years (PRF:33.2 ± 3; natural healing: 34.6 ± 4)	14/14	3 months	PRF	28	T: PRF (14) C:Natural (spontaneous) Healing (14)	Maxillary molar (13) Mandibular (15)	PRF is beneficial to increase the quality of the novel bone and enhance the rate of bone formation
Girish Kumar N	2018	India	RCT parallel-design	T1: 43.53 ± 15.84 T2: 47.76 ± 14.90 C: 42.1 ± 17.41	28/62	6 months	PRF	90	T1: PRF (30) T2:POP + PRF (30) C:Natural (spontaneous) Healing (30)	Anteriors (42) Premolars (27) Molars (31)	The PRF clinically contributes to better postoperative healing and minimal loss of alveolar width and height
Clark D	2018	USA	CCT parallel-design	58 ± SD	18/22	3 months	A-PRF	40	T1: A-PRF (10) T2:A-PRF + FDBA (10) T3: FDBA alone (10) C:Natural (spontaneous) Healing (10)	NG	A-PRF alone or augmented with FDBA is a suitable biomaterial for ridge preservation
Temmerman A	2016	Belgium	CCT split-mouth	54 ± 11	15/7	3 months	PRF	44	T: PRF (22) C:Natural (spontaneous) Healing (22)	Maxilla (18) Mandible (4)	PRF is beneficial to achieve preservation of horizontal and vertical ridge dimension

CCT, controlled clinical trial; RCT, randomized controlled trial; L-PRF, leukocyte and platelet-rich fibrin; HA, hyaluronic acid; DBBM, deproteinized bovine bone mineral (Bio-Oss® Collagen, Geistlich, Switzerland; Collagen, Bio-Gide®, Geistlich, Switzerland); ADDG, autogenous demineralized dentin graft; BBM/CM, bovine bone mineral/collagen membrane; FDBA/CM, freeze-dried bone allograft/collagen membrane; PRGF, plasma rich in growth; POP, plaster of Paris; NG, not given; SD, standard deviation.

### 3.2 The risk of bias

**Figure 2:**
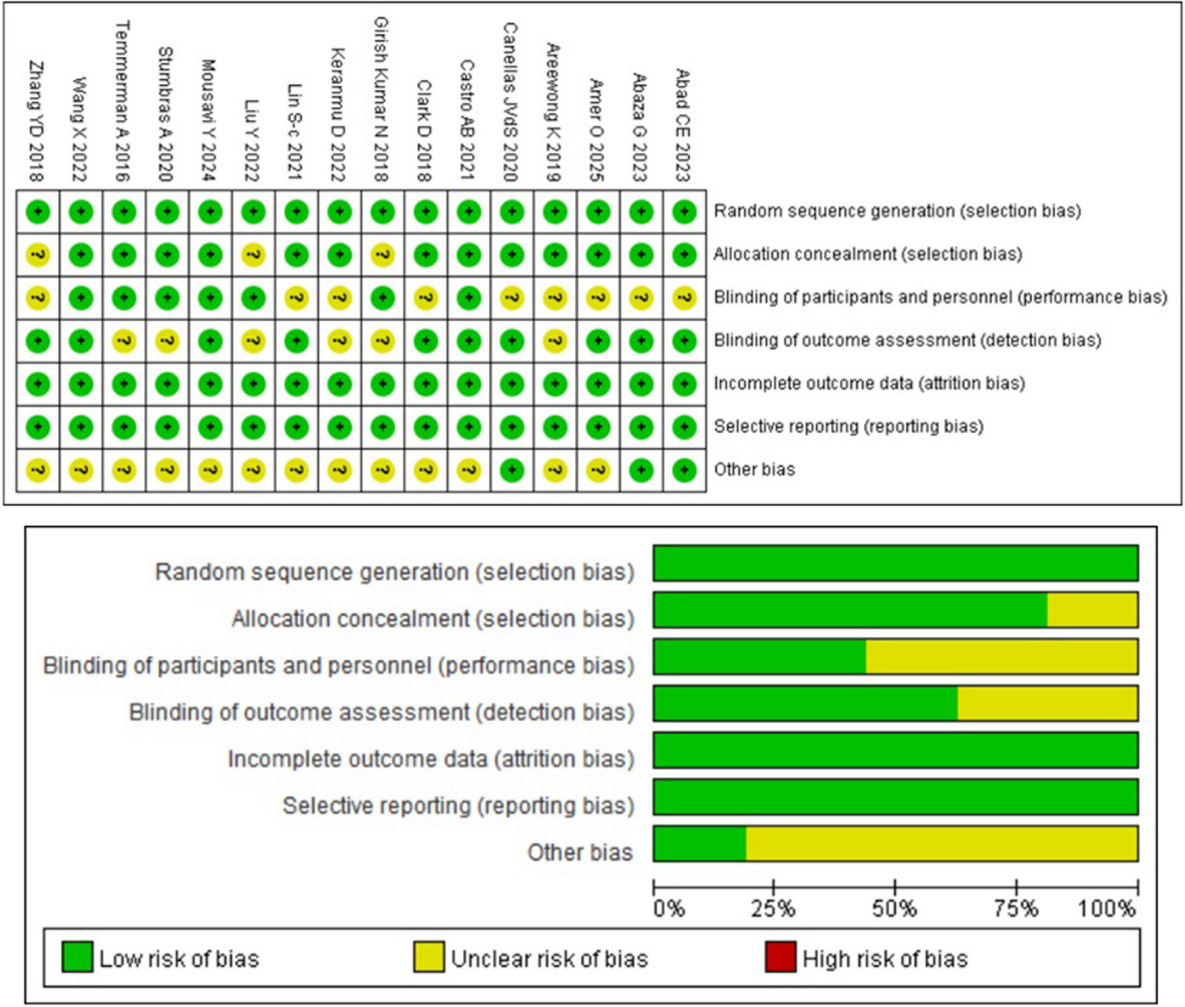
Main bias risk of the studies.

The results of the comparative studies for risk of bias assessment are shown in Figure 2, based on the RoB 2 tool. Six studies were classified as having a low risk of bias, and none were judged to be at high risk. The main sources of bias were performance bias and other bias. Three articles (23, 26, 31) did not report the methods used for random sequence generation. Performance bias was considered an unclear risk in nine articles (16–20, 22, 24, 25, 31), while detection bias was assessed as unclear in six articles (19, 23, 24, 26, 28, 29). The risk of other bias was unclear in 13 articles (18, 19, 21–31). No studies were found to have incomplete or selectively reported outcome data.

### 3.3 The synthesis of outcomes

The 16 included studies were categorized based on different measurement indices related to the use of APVBCs in the alveolar ridge. These included assessments of alveolar bone changes through radiographic and histological analyses. Based on the data from the included studies, we analyzed outcomes such as buccal/lingual ridge width reduction at 1, 3, and 5 mm below the bone crest, mean reduction in buccal ridge height, mean reduction in palatal/lingual ridge height, the rate of newly formed bone, and the percentage of remaining graft particles. In addition, meta-analyses were conducted to evaluate the effects of APVBCs alone and in combination with graft materials in socket preservation.

#### 3.3.1 The radiographic analysis of APVBCs and spontaneous healing in the buccal/lingual ridge width reduction at 1, 3, and 5 mm below the bone crest

CBCT scans were performed before tooth extraction and after the socket preservation procedure. Eight studies (16, 20–22, 27–30), including a total of 259 sockets, assessed changes in alveolar ridge dimensions 2–6 months after extraction. These measurements evaluated ridge width changes at 1, 3, and 5 mm below the alveolar crest, involving 130 sites in the autologous peripheral venous blood concentrate (APVBC) group and 129 sites in the spontaneous healing group.

**Figure 3:**
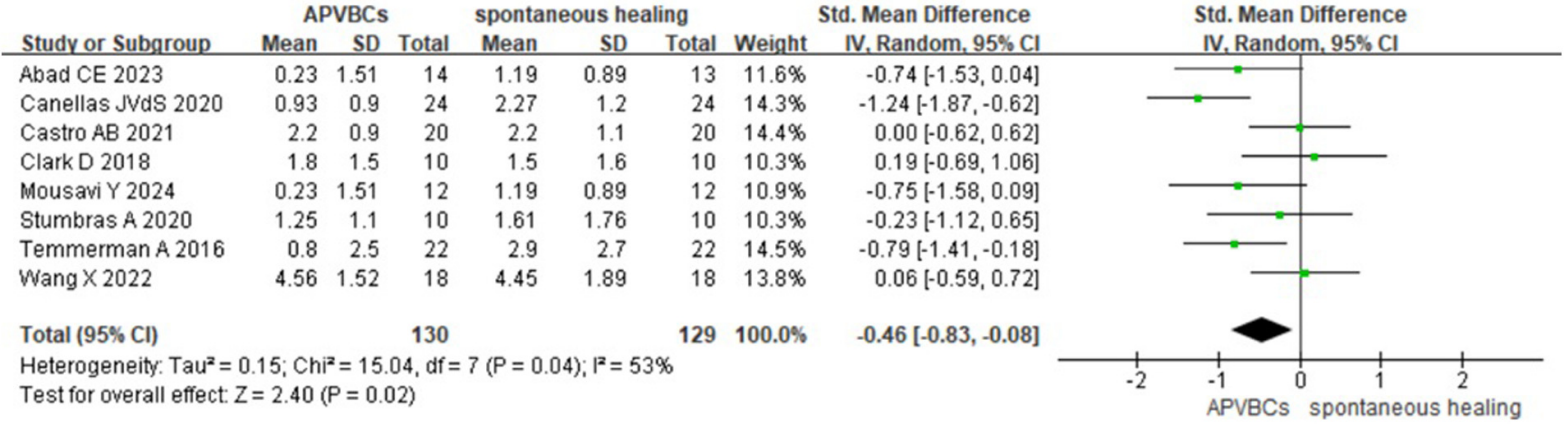
Radiographic analysis of APVBCs vs. spontaneous healing: buccal/lingual ridge width reduction at 1 mm below the bone crest.

The mean buccal/lingual alveolar bone width reduction at 1 mm below the bone crest was analyzed using a random-effects model. A statistically significant difference was observed between the APVBC and spontaneous healing groups, favoring the APVBC group (SMD: −0.46, 95% CI: −0.83 to −0.08; *p*= 0.02) (Figure 3). In addition, significant heterogeneity was observed in the analysis (*p* = 0.04, *I*^2^ = 53%).

**Figure 4:**
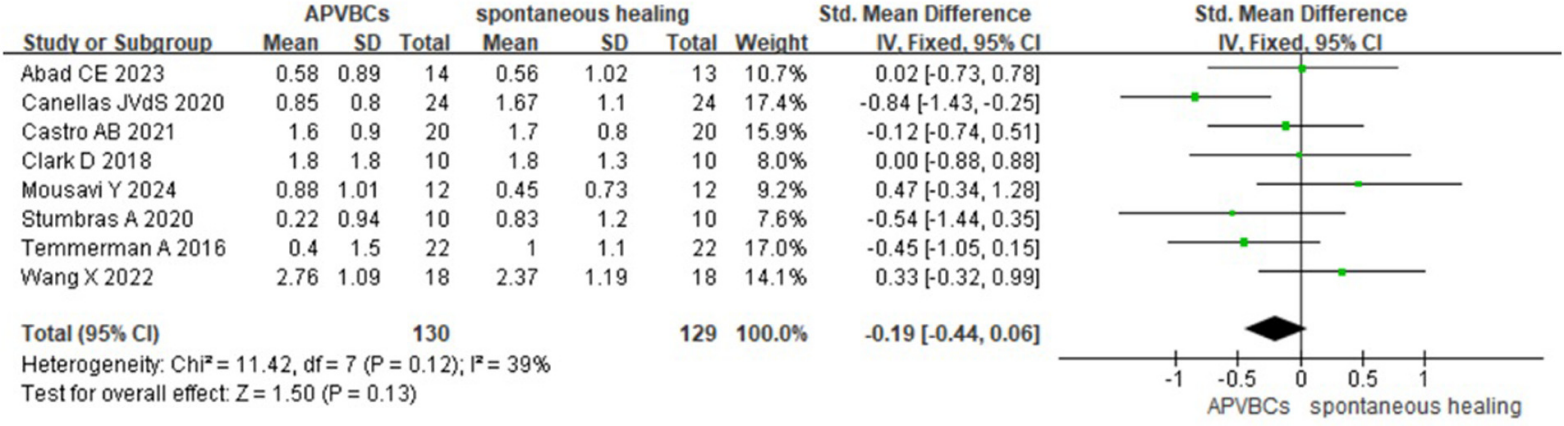
Radiographic analysis of APVBCs vs. spontaneous healing: buccal/lingual ridge width reduction at 3 mm below the bone crest.

For buccal/lingual ridge width reduction at 3 mm below the bone crest, a fixed-effects model was used. However, no significant difference was found between the APVBC and spontaneous healing groups (SMD: −0.19, 95% CI: −0.44 to 0.06; *p* = 0.13, *I*^2^ = 39%) (Figure 4).

**Figure 5:**
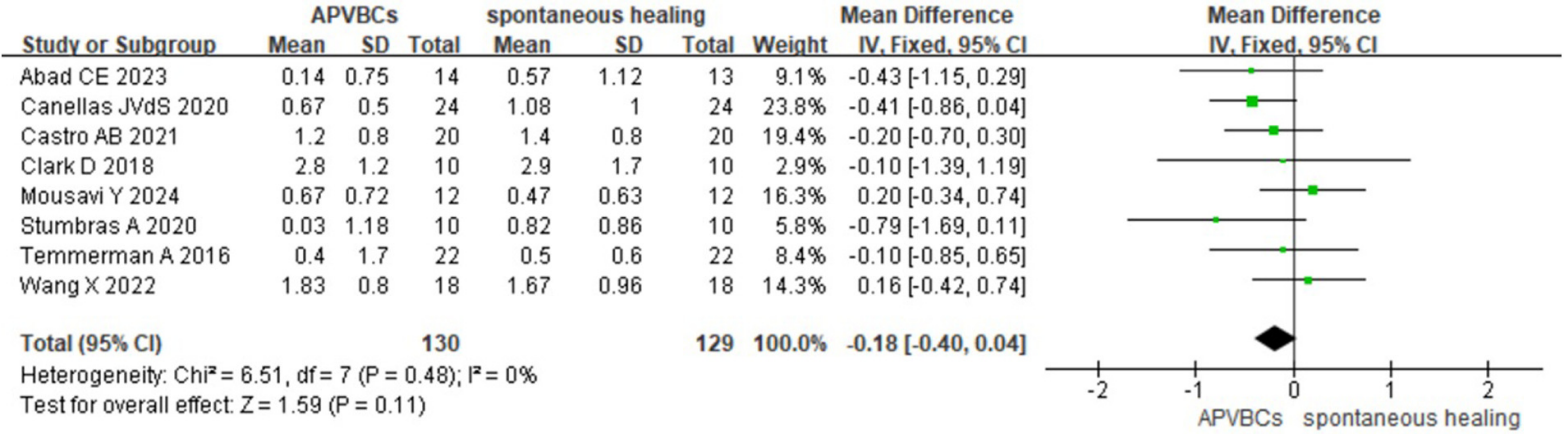
Radiographic analysis of APVBCs vs. spontaneous healing: buccal/lingual ridge width reduction at 5 mm below the bone crest.

Finally, the meta-analysis of buccal/lingual ridge width reduction at 5 mm below the bone crest also showed no statistically significant difference (SMD: −0.18, 95%CI: −0.40 to 0.04; *p* = 0.11) (Figure 5), with no heterogeneity observed across studies (*p* = 0.48, *I*^2^ = 0%).

#### 3.3.2 The radiographic analysis of APVBCs and spontaneous healing: mean reduction in buccal and palatal/lingual ridge heights

**Figure 6:**
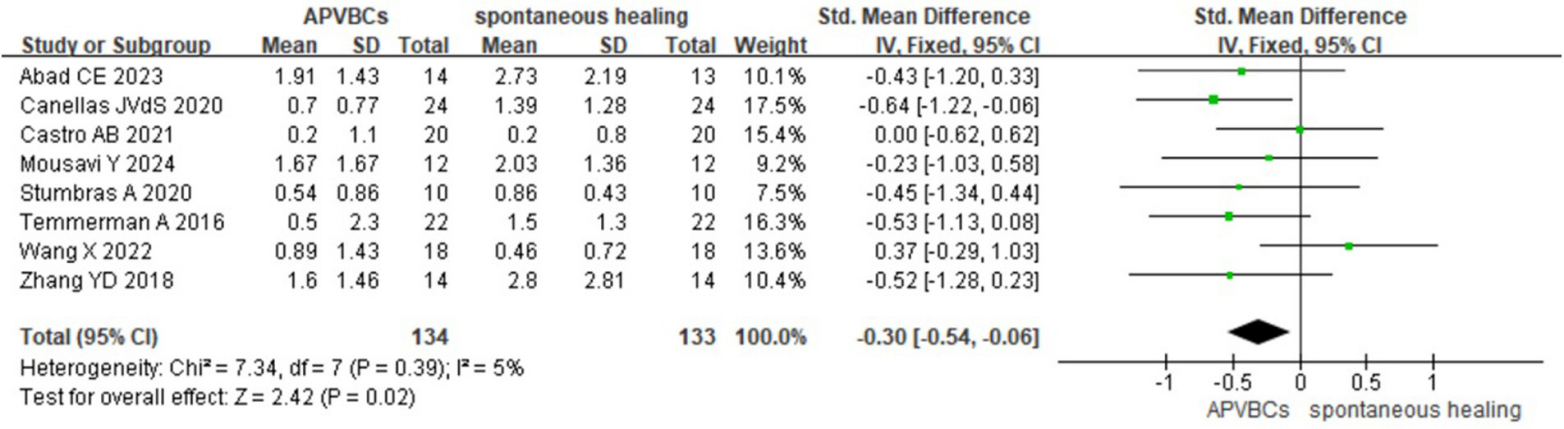
Radiographic analysis of APVBCs vs. spontaneous healing: mean reduction in buccal ridge heights.

Eight studies (16, 20, 21, 27–31) were included in the meta-analysis of changes in the vertical height of the buccal alveolar bone, encompassing a total of 267 extraction sockets. The results showed that vertical resorption of the buccal alveolar bone was significantly less in the APVBC group compared to spontaneous healing group, with a statistically significant difference. The meta-analysis findings are shown in Figure 6 (SMD: −0.30, 95% CI: −0.54 to −0.06; *p* = 0.02), and low heterogeneity was observed between studies (*p* = 0.39, *I*^2^ = 5%).

**Figure 7:**
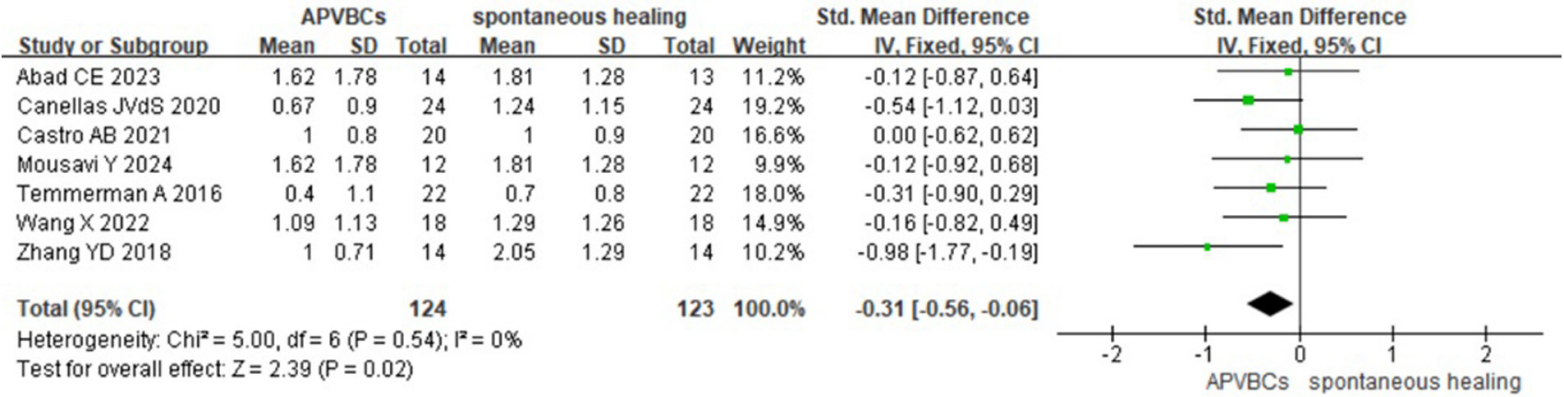
Radiographic analysis of APVBCs vs. spontaneous healing: mean reduction in palatal/lingual ridge heights.

A meta-analysis of the impact of APVBCs on mean reduction in palatal/lingual ridge height included seven studies (16, 20, 21, 27, 29–31), involving 124 sites in the APVBC group and 123 sites in the spontaneous healing group. The results demonstrated that the use of APVBCs during socket preservation had a statistically significant effect in reducing vertical resorption of the palatal/lingual alveolar bone (SMD: −0.31, 95% CI: −0.56 to −0.06; *p* = 0.02). No heterogeneity was observed among the studies (*p* = 0.54, *I*^2^ = 0%), as shown in Figure 7.

#### 3.3.3 The radiographic analysis of materials + APVBCs and materials: buccal/lingual ridge width reduction at 1, 3, and 5 mm below the bone crest

Four studies (18, 22, 24, 26) were included in the meta-analysis, involving 51 sites in the graft material + APVBC group and 51 sites in the material-only group. The results showed that the buccal/lingual ridge width reductions at 1, 3, and 5 mm below the bone crest in extraction sockets filled with graft materials combined with APVBCs were not significantly different from those in the graft material-only group.

**Figure 8:**
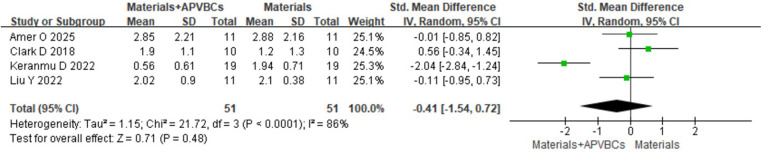
Radiographic analysis of materials + APVBCs vs. materials: buccal/lingual ridge width reduction at 1 mm below the bone crest.

**Figure 9:**
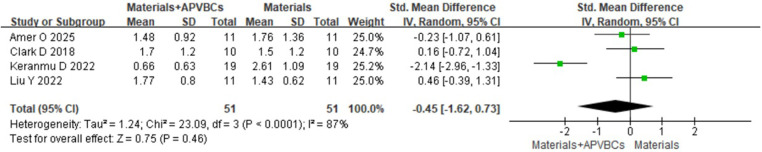
Radiographic analysis of materials + APVBCs vs. materials: buccal/lingual ridge width reduction at 3 mm below the bone crest.

**Figure 10:**
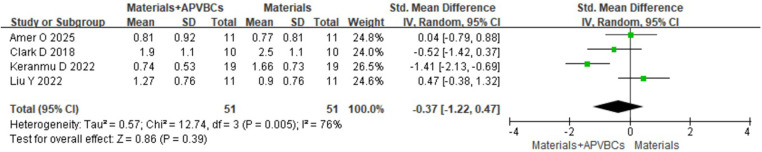
Radiographic analysis of materials + APVBCs vs. materials: buccal/lingual ridge width reduction at 5 mm below the bone crest.

At 1 mm below the bone crest, a random-effects model revealed no significant difference (SMD: −0.41, 95% CI: −1.54 to 0.72; *p* = 0.48) (Figure 8), with significant heterogeneity (*p* < 0.0001, *I*^2^ = 86%). At 3 mm below the bone crest, again using a random-effects model, there was no significant difference (SMD: −0.45, 95% CI:−1.62 to 0.73; *p* = 0.46) (Figure 9), with significant heterogeneity (*p* < 0.0001, *I*^2^ = 87%). At 5 mm below the bone crest, the analysis also showed no significant difference (SMD: −0.37, 95% CI: −1.22 to 0.47; *p* = 0.39) (Figure 10), with significant heterogeneity (*p* = 0.005, *I*^2^ = 76%).

#### 3.3.4 The radiographic analysis of materials + APVBCs and materials: mean reduction in buccal and palatal/lingual ridge heights

Only two studies (18, 24) were included in the radiographic analysis of mean reduction in buccal and palatal/lingual ridge heights, involving 30 sites in the materials + APVBC group and 30 sites in the materials-only group. Therefore, this section focuses on analyzing the efficacy of materials + APVBCs versus materials alone in socket preservation.

**Figure 11:**
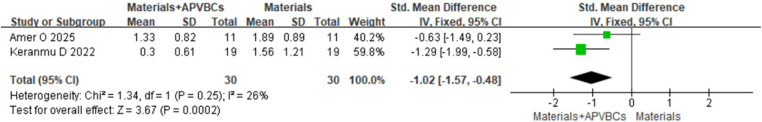
Radiographic analysis of materials + APVBCs vs. materials: mean reduction in buccal ridge heights.

**Figure 12:**
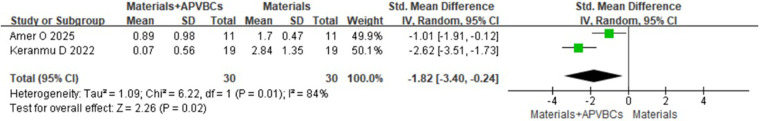
Radiographic analysis of materials + APVBCs vs. materials: mean reduction in palatal/lingual ridge heights.

The results showed that, compared with materials alone, the use of materials combined with APVBCs significantly reduced vertical resorption of the buccal alveolar bone (SMD: −1.02, 95% CI: −1.57 to −0.48; *p* = 0.0002) (Figure 11), with low heterogeneity (*p* = 0.25, *I*^2^ = 76%). Similarly, a significant reduction in vertical resorption of the palatal/lingual alveolar ridge was observed (SMD: −1.82, 95% CI: −3.40 to −0.24; *p* = 0.02) (Figure 12), with significant heterogeneity (*p* = 0.01, *I*^2^ = 84%).

#### 3.3.5 The histological evaluation of APVBCs versus spontaneous healing: the rate of newly formed bone

**Figure 13:**
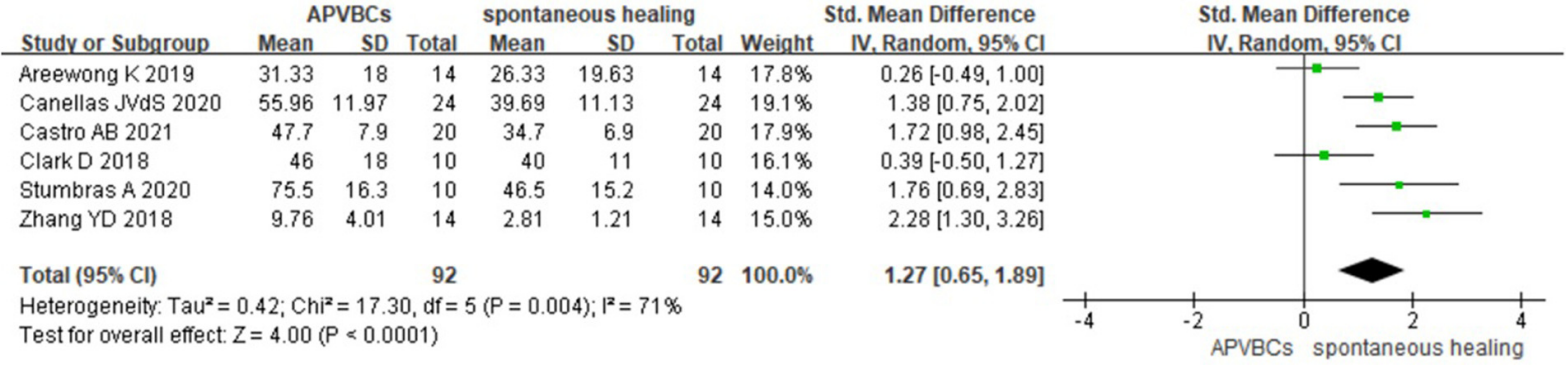
Histological evaluation of APVBCs vs. spontaneous healing: the rate of newly formed bone.

To evaluate the impact of APVBCs on the rate of newly formed bone, six studies were included (19–22, 28, 31), comprising a total of 184 sites across the APVBC and spontaneous healing groups. The results indicated that the use of APVBCs during socket preservation significantly increased the rate of newly formed bone (SMD: 1.27, 95% CI: 0.65–1.89; *p* < 0.0001) (Figure 13). However, significant heterogeneity was observed between the groups (*p* = 0.004, *I*^2^ = 71%).

#### 3.3.6 The histological evaluation of materials + APVBCs versus materials: the rate of newly formed bone and remaining graft particles

**Figure 14:**
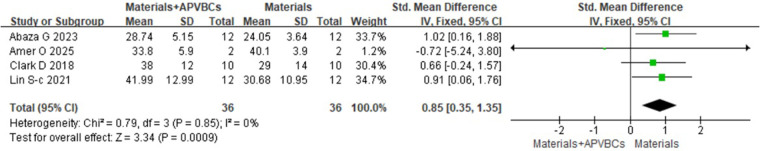
Histological evaluation of materials + APVBCs vs. materials: the rate of newly formed bone.

**Figure 15:**
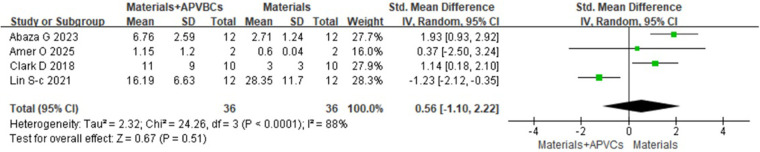
Histological evaluation of materials + APVBCs vs. materials: the rate of the remaining graft particles.

Four studies (17, 18, 22, 25), including a total of 72 sockets, reported the histological evaluation of APVBCs combed with graft materials in socket preservation, compared with graft materials alone. In the materials + APVBC group, more mature lamellar bone and active osteoblasts were observed at the border of the newly formed bone compared to the graft materials-only group. In addition, there was a significant increase in the percentage of newly formed bone in the materials + APVBC group (SMD: 0.85, 95% CI: 0.35–1.35; *p* = 0.0009) (Figure 14), with no heterogeneity observed (*p* = 0.85, *I*^2^ = 0%). However, the application of APVBCs combined with graft materials did not significantly influence the percentage of remaining graft particles compared to graft materials alone (SMD: 0.56, 95% CI: −1.10 to 2.22; *p* = 0.51) (Figure 15), and high heterogeneity was observed (*p* < 0.0001, *I*^2^ = 88%).

## 4 Discussion

Socket preservation surgery is commonly used to reduce the resorption of alveolar bone and soft tissue, aiming to maintain the contour of the alveolar ridge and ensure suitability for subsequent implant placement. APVBCs are platelet concentrates derived from the patient's peripheral blood, rich in growth factors and cytokines that promote tissue regeneration and wound healing (32). There are three generations of APVBCs: platelet-rich plasma (PRP), platelet-rich fibrin (PRF), and concentrated growth factors (CGFs). These concentrates contain a variety of growth factors, including vascular endothelial growth factor (VEGF), platelet-derived growth factors (PDGFs), epidermal growth factor (EGF), transforming growth factor β (TGF-β), BMPs, and insulin-like growth factor (IGF) (33).

Furthermore, APVBCs can play a scaffolding role in bone regeneration. Their biological scaffold features a highly biocompatible three-dimensional fiber network structure (34), which contains a significant concentration of aggregated platelets and encapsulates numerous growth factors. This fiber structure also retains various cell types while preventing rapid degradation (35). Due to the scaffold network, combining APVBCs with other osteogenic materials can significantly enhance regenerative potential and provide a more stable environment for new bone formation (36). In particular, the fiber structure in PRF has been shown – through fluorescence immunohistochemical analysis – to offer an ideal three-dimensional structure that supports bone healing and vascularization (37). A prospective study (38) reported that APVBCs combined with deproteinized bovine bone mineral (DBBM) appeared to promote effective horizontal bone gain in guided bone regeneration of the anterior maxilla and in immediate-loading full-arch rehabilitation. Similarly, Feng et al. (39) found that APVBC bone blocks combining PRF and DBBM for alveolar bone defects demonstrated superior mechanical and biological properties compared to either material used alone, as evidenced by encoding runt-related transcription factor 2 (RUNX2), alkaline phosphatase, collagen type I alpha1 (COL1A1), and osteocalcin (OCN). However, Dragonas et al. (40) concluded that combining APVBCs with DBBM did not improve new bone formation outcomes in maxillary sinus augmentation procedures, and none of the APVBCs were superior to any of the variables assessed. Therefore, there is still uncertainty about osteogenesis in the application of APVBCs.

The radiographic evaluation results were less conclusive when comparing APVBCs combined with graft materials to graft materials alone. Although there was a positive trend in analyzing the buccal/lingual ridge width and vertical resorption of the buccal and palatal/lingual alveolar bone in the APVBC group compared to spontaneous healing, statistically significant differences were only observed at 1 mm below the alveolar bone crest. No significant differences were found at 3 mm and 5 mm below the crest. Although alveolar bone height resorption was significantly reduced in all APVBC groups compared to controls, width changes at of 3 mm and5 mm below the alveolar bone crest did not show significant differences between groups. The lack of clear results regarding ridge width changes may be attributed to the differences in measurement methods. In addition, heterogeneity among the studies likely contributed to this difference. Due to uncertainty in the detection locations and the subjective nature of interpretation, gray value analysis of the alveolar bone was not included in all analyses.

Histomorphometric analysis has shown that pathological morphological changes related to successful socket preservation are associated with the use of APVBCs (24, 25). As shown in our meta-analysis, socket preservation using APVBCs showed significant advantages in promoting newly formed alveolar bone compared to natural healing, particularly in the materials + APVBC group, where no heterogeneity was observed (*p* = 0.85, *I*^2^ = 0%).

Zhu et al. (41) conducted a study evaluating APVBCs combined with deproteinized bovine bone mineral (DBBM) for simultaneous implant-guided bone regeneration at 6 months postoperatively. Their results demonstrated that, compared to the DBBM alone, the APVBC–DBBM mixture was more effective in reducing bone resorption, promoting bone reconstruction, and alleviating certain postoperative complications in implants with simultaneous GBR.

Ivanova and Chenchev (42) and Dwivedi and Kour (43) also reported that freeze-dried bone allograft (FDBA) combined with APVBCs, used as the sole grafting material for socket preservation, led to a significantly higher percentage of vital bone formation, as confirmed by histological analysis. Histological analysis using CD31 immunohistochemical staining showed that the density of new blood vessels – measured by positive CD31 expression – was significantly higher in the APVBC group (35.32 ± 3.47) compared to the Bio-Gide group (22.93 ± 4.42; *p* < 0.001) (44).

To achieve successful implant outcomes and long-term stability, sufficient bone volume is essential. Studies have suggested that the ideal bone graft material should not only provide osteoconduction but also promote osteoinduction and osteogenesis (45, 46). Therefore, identifying materials that enhance the osteoinductive potential of bone substitutes is crucial (47). DBBM has been shown to reliably support osteogenesis, and APVBCs share these characteristics, making them a valuable material for bone augmentation procedures (48, 49). The production of the APVBCs requires variable speeds to separate blood cells from fibrin-rich blocks that are denser and contain higher concentrations of growth factors.

Combining DBBM with APVBCs may help reduce bone grafting costs, shorten treatment duration, and create more favorable conditions for postoperative healing (50). As shown in our meta-analysis, more mature newly formed bone was observed in the APVBC group compared to the spontaneous healing group. Our findings also indicate that, when combined with graft materials, APVBCs significantly enhance new bone formation. However, the effect of APVBCs on the percentage of remaining graft particles requires further investigation through high-quality randomized controlled trials.

To the best of our knowledge, this is the first meta-analysis to evaluate both histological and radiological outcomes of APVBCs in socket preservation, specifically assessing the additional effect of APVBCs in enhancing bone tissue healing. Our findings support that socket preservation with APVBCs is an effective method for preventing alveolar bone resorption after tooth extraction.

However, there are some limitations in this meta-analysis. Despite similarities in study design, the included studies varied in terms of assessment timing, specific anatomical sites evaluated, testing methods, types of graft materials used, and centrifugation techniques for APVBCs. These variations contributed to a certain degree of heterogeneity across studies. Therefore, it is imperative to interpret the results with these factors in mind, even though all included studies provided valuable insights. There is still a need for more in-depth clinical RCT research.

## 5 Conclusions

In conclusion, this systematic review and meta-analysis evaluated the efficacy of APVBCs in socket preservation, particularly their radiological and histological effects on the alveolar ridge. The use of APVBCs in extraction sockets was shown to promote new bone formation and reduce vertical alveolar ridge resorption. However, their effect on reducing horizontal alveolar bone resorption was not significant, particularly when combined with graft materials and the remaining graft particles. Given the variability in findings and the heterogeneity among studies, the current evidence regarding the efficacy of APVBCs remains inconsistent. Further rigorous, clinical, randomized controlled trials are warranted to thoroughly investigate the full extent of clinical efficacy and mechanisms of APVBCs in socket preservation.

## Data Availability

The datasets presented in this study can be found in online repositories. The names of the repository/repositories and accession number(s) can be found in the article/Supplementary Material.
